# Supplementary material for: Associations of clothing size, adiposity and weight change with risk of postmenopausal breast cancer in the UK Women’s Cohort Study (UKWCS)
Source: BMJ Open. 2018 Sep 28;8(9):e022599. doi: 10.1136/bmjopen-2018-022599 (PMC6169772; doi:10.1136/bmjopen-2018-022599)
Supplement: Supplementary file 1 [file bmjopen-2018-022599supp001.pdf]

Supplementary Table:

Correlations between Skirt size, blouse size, waist circumference and BMI

	Skirt size	Blouse size	Waist Circumference	BMI
Skirt size	1.00			
Blouse size	0.91	1.00		
Waist circumference	0.76	0.73	1.00	
BMI	0.75	0.78	0.65	1.00

All p values <0.001
