# Supplementary material for: Associations of clothing size, adiposity and weight change with risk of postmenopausal breast cancer in the UK Women’s Cohort Study (UKWCS)
Source: BMJ Open. 2018 Sep 28;8(9):e022599. doi: 10.1136/bmjopen-2018-022599 (PMC6169772; doi:10.1136/bmjopen-2018-022599)
Supplement: Author's manuscript [file bmjopen-2018-022599.draft_revisions.pdf]

BMJ Open is committed to open peer review. As part of this commitment we make the peer review history of every article we publish publicly available.

When an article is published we post the peer reviewers' comments and the authors' responses online. We also post the versions of the paper that were used during peer review. These are the versions that the peer review comments apply to.

The versions of the paper that follow are the versions that were submitted during the peer review process. They are not the versions of record or the final published versions. They should not be cited or distributed as the published version of this manuscript.

BMJ Open is an open access journal and the full, final, typeset and author-corrected version of record of the manuscript is available on our site with no access controls, subscription charges or pay-per-view fees (http://bmjopen.bmj.com).

If you have any questions on BMJ Open's open peer review process please email info.bmjopen@bmj.com

BMJ Open

Associations of clothing size, adiposity and weight change with risk of post-menopausal breast cancer in the UK Women's Cohort Study (UKWCS)

Journal:	*BMJ Open*
Manuscript ID	bmjopen-2018-022599
Article Type:	Research
Date Submitted by the Author:	25-Feb-2018

Complete List of Authors:	Moy, Foong Ming; University of Malaya, Social & Preventive Medicine, Faculty of Medicine<br>Greenwood, Darren; University of Leeds, Division of Epidemiology & Biostatistics, School of Medicine<br>Cade, Janet; University of Leeds, Nutritional Epidemiology Group, School of Food Science and Nutrition

Keywords:	blouse size, skirt size, weight change, post-menopausal breast cancer, UK Women Cohort

SCHOLARONE™ Manuscripts

For peer review only

Associations of clothing size, adiposity and weight change with risk of post-menopausal breast cancer in the UK Women's Cohort Study (UKWCS)

Foong Ming Moy [1,2], Darren C Greenwood[3], Janet E Cade[2]

[1]Julius Centre University of Malaya, Department of Social & Preventive Medicine, Faculty of Medicine, University of Malaya, 50603 Kuala Lumpur, Malaysia

[2]Nutritional Epidemiology Group, School of Food Science & Nutrition, University of Leeds, Leeds, LS2 9JT, United Kingdom

[3] Division of Epidemiology & Biostatistics, School of Medicine, University of Leeds, Leeds, LS2 9NL, United Kingdom

Corresponding author:

Dr Foong Ming Moy,

Julius Centre University of Malaya, Department of Social & Preventive Medicine, Faculty of Medicine, University of Malaya, Kuala Lumpur, Malaysia

Email: moyfm@ummc.edu.my

For peer review only

For peer review only - http://bmjopen.bmj.com/site/about/guidelines.xhtml

Abstract:

Objectives: Breast cancer is associated with overweight and obesity after menopause. However, clothing size as a proxy of adiposity in predicting post-menopausal breast cancer is not widely studied. We aimed to explore the relationships between post-menopausal breast cancer risk with adipose indicators (including clothing sizes) and weight change over adulthood.

Design: Prospective cohort study

Setting: England, Wales and Scotland

Participants: 17,781 post-menopausal women from the UK Women's Cohort Study

Primary outcome measure: Incident cases of malignant breast cancers (ICD9 Code 174 and ICD 10 Code C50)

Results: From 282,277 person years follow up, there were 946 incident breast cancer cases with an incidence rate of 3.35 per 1,000 women. BMI (HR: 1.04; 95% CI: 1.02, 1.07), blouse size (HR: 1.10; 1.03, 1.18), waist circumference (HR: 1.07; 1.01, 1.14) and skirt size (HR: 1.14 (1.06, 1.22) had positive associations with post-menopausal breast cancer after adjustment for potential confounders. Increased weight over adulthood (HR: 1.02; 1.01, 1.03) was also associated with increased risk for post-menopausal breast cancer.

Conclusions: Blouse and skirt sizes can be used as adipose indicators in predicting post-menopausal breast cancer. Maintaining healthy body weight over adulthood is an effective measure in the prevention of post-menopausal breast cancer.

Strengths and limitations of this study:

This study may be the first to report that both blouse and skirt sizes predict post-menopausal breast cancer risk equally as well as other more invasive measures of body size.

The large study sample with long follow up duration and linkage of participants' ID with the National Statistics National Health Service central register provides accurate information on breast cancer diagnosis.

All adipose indicators, blouse and skirt sizes were self-reported.

We were unable to evaluate in more detail the effect of weight gain at different ages.

Breast tumour characteristics were not available and we could not explore whether our findings may differ by breast cancer subtypes.

Keywords: blouse size, skirt size, adiposity, weight change, post-menopausal breast cancer, UK Women Cohort

For peer review only - http://bmjopen.bmj.com/site/about/guidelines.xhtml

Introduction:

Breast cancer is the most common cancer among women worldwide, accounting for 25% of new cases [1]. In the UK, it is also the most common cancer among women with around 54,800 cases diagnosed in 2014 [2]. The prevalence of obesity in the UK has increased from 15% in 1993 to 27% in 2015, and 57% of the women were overweight or obese [3]. Obesity is associated with increased risks of chronic diseases and certain cancers [4 5]. There is strong evidence that obesity and weight gain over adult life are associated with increased risk of post-menopausal breast cancer [6 7]. Maintaining healthy weight is a key recommendation to prevent post-menopausal breast cancer[6].

To date, many adipose indicators namely weight, body mass index (BMI), waist circumference, waist to hip ratio are used in research and health education programmes. Body Mass Index (BMI) is an established indicator for general obesity, while central obesity is commonly measured using waist circumference. Among post-menopausal women, higher BMI and larger waist circumference during adulthood; and weight gain over adulthood are associated with increased breast cancer risk [6].

Skirt or trouser size [8-10] have been suggested to be used as proxy for central obesity. Skirt size appears to predict cancer risk independently of BMI [10]. Change in skirt size was also found to be predictive of post-menopausal breast cancer [11]. However, blouse size has not been explored as a proxy for general obesity in predicting post-menopausal breast cancer. In view of the limited evidence of using clothing size as an adiposity indicator in predicting post-menopausal breast cancer risk, and the ease of self-reporting of these measures, we have used data from the UK Women's Cohort Study (UKWCS) to explore the relationships between post-menopausal breast cancer risk with various adipose indicators (including skirt and blouse sizes); as well as weight change over adulthood.

For peer review only

For peer review only - http://bmjopen.bmj.com/site/about/guidelines.xhtml

Materials and methods:

Study design and population

The recruitment of participants and characteristics of the UKWCS have been described in detail previously [12]. In brief, a total of 35 792 women were recruited between 1995 and 1998 via a postal questionnaire. They were aged 35 to 69 years at baseline and were living in England, Wales and Scotland. In total, 17 781 women were post-menopausal and 15 951 women were pre-menopausal at baseline. Post-menopausal women were those who had not had a natural menstrual period in the last 12 months, or were older than 50 years old and currently on hormone replacement therapy (HRT), or with a previous hysterectomy and HRT. Where these data were missing, if the woman was aged more than 50 years she was also counted as menopausal. Only post-menopausal women were included in the current analyses.

Ethics clearance

A total of 174 local research ethics committees were contacted and permission to carry out the baseline UKWCS study was obtained [13]. Participants had consented to the use of information gathered at baseline, future phases and cancer registries for research purposes provided that confidentiality was maintained. The National Research Ethics Committee for Yorkshire and the Humber, Leeds East have now taken on responsibility for the on-going cohort[14].

Case definition and ascertainment

All women were flagged for death and cancer registration on the office of National Statistics National Health Service central register. Causes of death and cancer diagnosis were coded in the International Classification of Diseases (ICD) 9 and 10. All malignant breast cancers (ICD9 Code 174 and ICD 10 Code C50) registered after the participants returned their questionnaires were taken as incident cases. Prevalent cancers identified from the questionnaires and incident cases identified within one year from the date of questionnaire returned were excluded to rule out reverse causation since undiagnosed breast cancer may influence weight.

Assessment of adipose indicators

Anthropometric measures such as weight, height, waist and hip circumference at enrolment; and weight at age 20 years were self-recorded. Body mass index (BMI) was categorised into groups of underweight, normal weight, overweight and obese according to the WHO criteria [15]. Weight change over adulthood was computed by weight at enrolment minus weight at 20 years old. Weight change was further categorised into stable weight ($\pm$ 2kg), weight loss (2 to 4.9kg and $\geq$ 5kg) and weight gain (2 to 9.9kg, 10 to 19.9kg and $\geq$20kg). Skirt size and blouse size at enrolment were also reported. The clothing sizes ranged from 6 to 26 with the increase in units of 2.

For peer review only - http://bmjopen.bmj.com/site/about/guidelines.xhtml

Other confounding factors

Socio-economic characteristics, dietary pattern (meat eaters, fish eaters or vegetarian), lifestyle (smoking, alcohol consumption, physical activity etc), reproductive history, information on past health experience, sibling and parental health were reported at baseline.

Statistical analysis

The follow up (person-years) for each participant was counted from the beginning of the study until the date of breast cancer diagnosis or the censor date (1 April 2014), whichever occurred first. The median follow up was 16.9 years. The relationships between adipose indicators and weight change; and post-menopausal breast cancer were explored using Cox's proportional hazards regression. Hazard ratios with 95% confidence interval were reported. Linear trends, using the exposure categories as a continuous variable were also presented. Potential confounders such as age, highest education levels achieved, time spent walking (hours/day), family history of breast cancer, oral contraceptive use, hormone replacement therapy (HRT) use, parity, age at first birth, age of menarche, and dietary pattern were included in the fully adjusted models. In addition, BMI at 20 years was also adjusted for all adipose indicators, except BMI at 20 years. All statistical analyses were conducted using Stata version 14.

Results

After excluding the prevalent cases at baseline and incident cases developed within one year of questionnaire returned, 17,715 post-menopausal women were included in this analysis. From 282,277 person years follow up, there were 946 incident breast cancer cases with an incidence rate of 3.35 per 1,000 women.

The mean (SD) age of the post-menopausal participants at enrolment was 58.8 (7.5) years. The majority were white (99.1%); about one fifth had tertiary qualifications; 10% were smokers; 11% self-reported being on a vegetarian diet; 3% reported a family history of breast cancer; about 41% had ever taken HRT; 52% ever used OCP and 17% were nulliparous. The mean (SD) BMI at enrolment and at 20 years of age were 24.9(4.3) kg/m^2 and 21.5(2.9) kg/m^2 respectively (Table 1). The correlation between skirt size and blouse size was very high (r=0.91, p<0.001) while the correlation between BMI and waist circumference was only moderate (r=0.65, p<0.001). Skirt size was highly correlated with waist circumference (r=0.76, p<0.0001) while blouse size was highly correlated with BMI (0.78, p<0.0001).

Table 1: Descriptive characteristics of post-menopausal women

Incidence rates (per 1,000 women) for post-menopausal breast cancer increased by BMI categories at enrolment, BMI categories at 20 years old and weight change (from 20 years old to enrolment age) (Table 2). Obese women at recruitment had the highest incidence rates while women with the highest weight loss from age 20 had the lowest incidence rate of post-menopausal breast cancer.

Table 2: Incidence rates of post-menopausal breast cancer by BMI groups and groups of weight change

For peer review only - http://bmjopen.bmj.com/site/about/guidelines.xhtml

After adjustment for potential confounders, adipose indicators at enrolment namely BMI, waist circumference and clothing sizes (blouse and skirt sizes) were associated with increased risk for post-menopausal breast cancer (Table 3). Post-menopausal women who were overweight (HR: 1.34, 95% CI: 1.09, 1.65) or obese (HR: 1.27, 95% CI: 0.91, 1.76) had higher risks of breast cancer compared to those with normal weight at enrolment. There was no association observed for women in the underweight category. No association was observed for BMI at 20 years with post-menopausal breast cancer risk.

Table 3: Crude and adjusted hazard ratio (HR) of general and central adiposity indicators

BMI and blouse size (which indicate general obesity) as well as waist circumference and skirt size (which indicate central obesity) had positive association with post-menopausal breast cancer. There was an increase of 3% in HR for every unit increase in BMI and 6% in HR for every 5cm increase in waist circumference after adjustment for confounders. There was an additional 1% increase in HRs after additionally adjusted for BMI at 20 years for both BMI and waist circumference. For every 2-unit increase in blouse and skirt sizes (as UK clothes size increased by 2 units), the HRs for blouse and skirt sizes were 1.08 (95% CI: 1.01, 1.14) and 1.12 (95% CI: 1.06, 1.19) respectively. The HRs increased to 1.10 (95% CI: 1.03, 1.18) and 1.14 (95% CI: 1.06, 1.22) for blouse and skirt sizes respectively after additional adjustment for BMI at 20 years of age.

Weight change over adulthood was associated with increased risk for post-menopausal breast cancer (HR: 1.02, 95% CI: 1.01, 1.03). For every 1kg weight change, there was a corresponding 2% risk increase after adjusting for confounders, similar results were observed when weight change was additionally adjusted for BMI at 20 years (Table 4). There was an increasing trend in associated risks with post-menopausal breast cancer (P for trend <0.001) when weight change was categorised into groups of weight loss, stable weight and increased weight. Using stable weight as the reference category, women who experienced weight loss had lower risk, while those with weight gain had higher risk for post-menopausal breast cancer.

Table 4: Crude and adjusted Hazard Ratio (HR) of weight change from age 20 years

For peer review only

For peer review only - http://bmjopen.bmj.com/site/about/guidelines.xhtml

Discussion

Our results demonstrated that clothing size such as blouse and skirt sizes can be used as adipose indicators in predicting post-menopausal breast cancer. Trouser and skirt size have been reported to predict cancer risks independent of BMI [10] while skirt size changes between 20 years of age and postmenopausal-age was reported to be associated with post-menopausal breast cancer [11]. However, blouse size has not been explored as a proxy for general obesity in predicting post-menopausal breast cancer. Our study may be the first to provide evidence that larger blouse size is associated with increased risk of post-menopausal breast cancer.

Skirt size and waist circumference were highly correlated and performed equally well in predicting post-menopausal breast cancer risk. Our findings provide additional evidence of using skirt size as a proxy for central obesity in predicting post-menopausal breast cancer. On the other hand, there was no previous report on blouse size associated with increased risk of post-menopausal breast cancer. Blouse size could serve as an indicator for general obesity as it has a strong correlation with BMI. Blouse size also performed as well as BMI in predicting breast cancer among post-menopausal women. A previous study conducted in the UK reported that among 362 respondents, only 3% reported a clothing size different from their clothing labels [9]. This indicated that self-reported clothing size is reliable. Blouse and skirt sizes which are simple and non-invasive self-reported measures could be promoted as proxies for adipose indictors. From a public health perspective, these findings are significant when anthropometric measurements are not available, or challenging in their measurements due to extreme obesity [10].

Overweight and obese women at enrolment had up to 32% increased risk of post-menopausal breast cancer. The association between overweight / obesity with post-menopausal breast cancer remained after further adjustment for BMI at 20 years. Women who gained the most weight since the age of 20 years old, had almost 30% increased risk of post-menopausal breast cancer after adjustment for potential confounders and BMI at 20 years. Similar findings have been reported by other researchers [16-19]. These results suggest that regardless of BMI in early adulthood, being obese in later life or gaining weight along the way increases deposition of adipose tissue. After menopause, adipose tissue is the major location for the synthesis of oestrogens. Circulating oestrogen are higher in obese than in slim post-menopausal women [19]. Obesity also contributes to inflammatory changes leading to macrophage polarization and altered adipokine profile [20]. These mechanisms may explain the association which links obesity and post-menopausal breast cancer together.

Consistent with previous literature [6 11 21]; waist circumference as an indicator for central obesity was found to be positively associated with post-menopausal breast cancer. The association remained after adjustment for potential confounders and BMI at 20 years. This may indicate that regardless of being obese in early adulthood or at later life, being centrally obese in later life increases the risk of post-menopausal breast cancers. Central obesity is associated with increased insulin levels and insulin like growth factors which may stimulate the growth of breast cancer cells and neoplastic degeneration [22].

According to the latest report (2017) from the World Cancer Research Fund International / American Institute for Cancer Research, being overweight or obese during young adulthood decreases the risk of

For peer review only - http://bmjopen.bmj.com/site/about/guidelines.xhtml

post-menopausal breast cancer [6]. Low levels of adiposity in the mammary gland may alter breast tissue maturation, making breast tissue more susceptible to carcinogenic stimuli among leaner women [23]. Sex hormone levels may also explain the inverse relationship between early life adiposity and breast cancer risk. Obese young women may be more likely to experience anovulation and lower levels of progesterone and oestrogen [24], which could render a protective effect for risk of breast cancer after menopause. However, we did not find BMI at 20 years to be positively associated with post-menopausal breast cancer incidence, as also reported by other cohort studies [16 23 25].

There are a few limitations which need to be considered while interpreting the results. All adipose indicators, blouse and skirt sizes were self-reported. Some overweight or obese women may be more likely to under-report their weight compared to normal weight participants. On the other hand, blouse and skirt sizes offer an objective standard as a proxy to report overweight / obesity, which are potentially less liable for misreporting. We were unable to evaluate in more detail the effect of weight gain at different ages, other than that of weight at age 20 years and weight at enrolment. Breast tumour characteristics were not available and we could not explore whether our findings may differ by breast cancer subtypes. On the other hand, we believe we are the first to report that both blouse and skirt sizes predict post-menopausal breast cancer risk equally as well as other more invasive measures of body size. In addition, our study sample is large with long follow up duration. The linkage of participants' ID with the National Statistics National Health Service central register provides accurate information on breast cancer diagnosis.

Conclusion

Overweight/obesity (BMI and blouse size), central obesity (waist circumference and skirt size) and weight change during adulthood are associated with increased risk of post-menopausal breast cancer. Clothing size such as skirt and blouse sizes can be used as a proxy for adipose indicators in predicting post-menopausal breast cancer. Maintaining healthy body weight over adulthood is an effective measure in the prevention of post-menopausal breast cancer.

For peer review only

Acknowledgements:

We would like to acknowledge all women who participated in our study. The support from the Nutritional Epidemiology Group, University of Leeds is also acknowledged.

Funding:

JC was funded by a Medical Research Council grant no: MR/L02019X/1. The UKWCS was originally funded by the World Cancer Research Fund. Other support has been received from the Food Standards Agency (grant NO5 023), the Medical Research Council (ref: G1100235/1), the Wellcome Trust (Fellowship WT87789), the Economic and Social Research Council, Kelloggs Sales and Marketing UK Ltd.

Contributors:

Conception and design: FMM, JEC and DCG. Analysis and interpretation: FMM, JEC and DCG. Data collection: JEC and DCG. Manuscript preparation: FMM, JEC and DCG. Overall responsibility: FMM

For peer review only - http://bmjopen.bmj.com/site/about/guidelines.xhtml

Disclaimer:

The authors are responsible for the content and writing of this article. JEC had full access to all of the data in the study and takes responsibility for the integrity of the data. FMM, JEC and DCG takes responsibility of the accuracy of data analysis.

Competing interests:

None declared

For peer review only

For peer review only - http://bmjopen.bmj.com/site/about/guidelines.xhtml

References:

1. IARC, WHO. GLOBOCAN 2012: Estimated cancer incidence, mortality and prevalence worldwide in 2012. Secondary GLOBOCAN 2012: Estimated cancer incidence, mortality and prevalence worldwide in 2012 2012. http://globocan.iarc.fr/Pages/fact_sheets_cancer.aspx.
2. Cancer Research UK Breast cancer statistics : Breast cancer incidence. Secondary Breast cancer statistics : Breast cancer incidence 2014. http://www.cancerresearchuk.org/health-professional/cancer-statistics/statistics-by-cancer-type/breast-cancer.
3. NHS Digital. Statistics on Obesity, Physical Activity and Diet, England 2017: Report, 2017.
4. Wang J, Yang DL, Chen ZZ, et al. Associations of body mass index with cancer incidence among populations, genders, and menopausal status: A systematic review and meta-analysis. Cancer epidemiology 2016;**42**:1-8 doi: 10.1016/j.canep.2016.02.010[published Online First: Epub Date]|.
5. Renehan AG, Soerjomataram I. Obesity as an Avoidable Cause of Cancer (Attributable Risks). Recent results in cancer research Fortschritte der Krebsforschung Progres dans les recherches sur le cancer 2016;**208**:243-56 doi: 10.1007/978-3-319-42542-9_13[published Online First: Epub Date]|.
6. World Cancer Research Fund International / American Institute for Cancer Research. Continuous Update Project Report: Diet, nutrition, physical activity and breast cancer. Secondary Continuous Update Project Report: Diet, nutrition, physical activity and breast cancer 2017. wcrf.org/breast-cancer-2017.
7. Tamimi RM, Spiegelman D, Smith-Warner SA, et al. Population Attributable Risk of Modifiable and Nonmodifiable Breast Cancer Risk Factors in Postmenopausal Breast Cancer. American journal of epidemiology 2016;**184**(12):884-93 doi: 10.1093/aje/kww145[published Online First: Epub Date]|.
8. Battram DS, Beynon C, He M. The reliability and validity of using clothing size as a proxy for waist circumference measurement in adults. Applied Physiology, Nutrition, and Metabolism 2011;**36**(2):183-90 doi: 10.1139/h11-001[published Online First: Epub Date]|.
9. Han TS, Gates E, Truscott E, et al. Clothing size as an indicator of adiposity, ischaemic heart disease and cardiovascular risks. J Hum Nutr Diet 2005;**18**(6):423-30
10. Hughes LA, Schouten LJ, Goldbohm RA, et al. Self-reported clothing size as a proxy measure for body size. Epidemiology (Cambridge, Mass) 2009;**20**(5):673-6 doi: 10.1097/EDE.0b013e3181a66eb5[published Online First: Epub Date]|.
11. Fourkala EO, Burnell M, Cox C, et al. Association of skirt size and postmenopausal breast cancer risk in older women: a cohort study within the UK Collaborative Trial of Ovarian Cancer Screening (UKCTOCS). BMJ open 2014;**4**(9):e005400 doi: 10.1136/bmjopen-2014-005400[published Online First: Epub Date]|.
12. Cade JE, Burley VJ, Alwan NA, et al. Cohort Profile: The UK Women's Cohort Study (UKWCS). International journal of epidemiology 2017;**46**(2):e11 doi: 10.1093/ije/dyv173[published Online First: Epub Date]|.
13. von Koerber K, Bader N, Leitzmann C. Wholesome Nutrition: an example for a sustainable diet. Proc Nutr Soc 2017;**76**(1):34-41 doi: 10.1017/s0029665116000616[published Online First: Epub Date]|.
14. Nelson ME, Hamm MW, Hu FB, et al. Alignment of Healthy Dietary Patterns and Environmental Sustainability: A Systematic Review. Adv Nutr 2016;**7**(6):1005-25 doi: 10.3945/an.116.012567[published Online First: Epub Date]|.
15. WHO. Preventing and managing the global epidemic. Report of a WHO Consultation on Obesity. . Geneva: World Health Organisation, 1998.

For peer review only - http://bmjopen.bmj.com/site/about/guidelines.xhtml

16. Han X, Stevens J, Truesdale KP, et al. Body mass index at early adulthood, subsequent weight change and cancer incidence and mortality. International journal of cancer 2014;**135**(12):2900-9 doi: 10.1002/ijc.28930[published Online First: Epub Date]|.
17. Alsaker MD, Janszky I, Opdahl S, et al. Weight change in adulthood and risk of postmenopausal breast cancer: the HUNT study of Norway. British journal of cancer 2013;**109**(5):1310-7 doi: 10.1038/bjc.2013.403[published Online First: Epub Date]|.
18. Rosner B, Eliassen AH, Toriola AT, et al. Weight and weight changes in early adulthood and later breast cancer risk. International journal of cancer 2017;**140**(9):2003-14 doi: 10.1002/ijc.30627[published Online First: Epub Date]|.
19. Welti LM, Beavers DP, Caan BJ, et al. Weight Fluctuation and Cancer Risk in Postmenopausal Women: The Women's Health Initiative. Cancer epidemiology, biomarkers & prevention : a publication of the American Association for Cancer Research, cosponsored by the American Society of Preventive Oncology 2017;**26**(5):779-86 doi: 10.1158/1055-9965.epi-16-0611[published Online First: Epub Date]|.
20. Perez-Hernandez AI, Catalan V, Gomez-Ambrosi J, et al. Mechanisms linking excess adiposity and carcinogenesis promotion. Frontiers in endocrinology 2014;**5**:65 doi: 10.3389/fendo.2014.00065[published Online First: Epub Date]|.
21. Harvie M, Hooper L, Howell AH. Central obesity and breast cancer risk: a systematic review. Obesity reviews : an official journal of the International Association for the Study of Obesity 2003;**4**(3):157-73
22. Rose DP, Komninou D, Stephenson GD. Obesity, adipocytokines, and insulin resistance in breast cancer. Obesity reviews : an official journal of the International Association for the Study of Obesity 2004;**5**(3):153-65 doi: 10.1111/j.1467-789X.2004.00142.x[published Online First: Epub Date]|.
23. Suzuki R, Iwasaki M, Inoue M, et al. Body weight at age 20 years, subsequent weight change and breast cancer risk defined by estrogen and progesterone receptor status--the Japan public health center-based prospective study. International journal of cancer 2011;**129**(5):1214-24 doi: 10.1002/ijc.25744[published Online First: Epub Date]|.
24. Pasquali R, Pelusi C, Genghini S, et al. Obesity and reproductive disorders in women. Human reproduction update 2003;**9**(4):359-72
25. Krishnan K, Bassett JK, MacInnis RJ, et al. Associations between weight in early adulthood, change in weight, and breast cancer risk in postmenopausal women. Cancer epidemiology, biomarkers & prevention : a publication of the American Association for Cancer Research, cosponsored by the American Society of Preventive Oncology 2013;**22**(8):1409-16 doi: 10.1158/1055-9965.EPI-13-0136[published Online First: Epub Date]|.

For peer review only

For peer review only - http://bmjopen.bmj.com/site/about/guidelines.xhtml

For peer review only

For peer review only - http://bmjopen.bmj.com/site/about/guidelines.xhtml

Table 1: Descriptive characteristics of post-menopausal women (n=17,715)

	Total	n (%)
Age (mean ± SD) years	17,686	58.8±7.5
Highest education achieved	15,521	
No education		3,950 (25.5)
O-level		4,682 (30.2)
A-level		3,700 (23.8)
Degree		3,189 (20.6)
White	17,149	16, 995 (99.1)
Food groups	15,602	
Meat eaters		12,175 (78.0)
Fish eaters		1,700 (10.9)
Vegetarians		1,727 (11.1)
Smokers	17,714	1,758 (9.9)
Walking (mean ± SD) (hours /day)	15,866	0.94±0.77
Family history of breast cancer	17,715	539 (3.0)
Ever taken HRT	17,031	7,045 (41.4)
Ever used OCP	17,362	8,997 (51.8)
Parity	17,292	
0		2,937 (16.9)
1-2		8,685 (50.2)
3-4		5,129 (29.7)
>=5		541 (3.1)
Age of menarche (mean ± SD) (years)	17,247	12.9±1.6
Age of first birth (mean ± SD) (years)	14,336	25.3±4.4
BMI at enrolment (mean ± SD) (kg/m^2)	17,029	24.9±4.3
Waist circumference (mean ± SD) (cm)	14,491	75.3±9.7
BMI at 20 years (mean ± SD) (kg/m^2)	16,077	21.5±2.9
Weight at 20 years (mean ± SD) (kg)	16,388	57.5±8.3

For peer review only

Table 1: Descriptive characteristics of post-menopausal women (n=17,715) (Continued..)

BMI categories at enrolment	17,029	
Underweight (<18.5 kg/m^2)		327 (1.9)
Normal weight (18.5-24.9 kg/m^2)		9,816 (57.6)
Overweight (25.0-29.9 kg/m^2)		4,936 (29.0)
Obese ($\geq$ 30.0kg/m^2)		1,950 (11. 5)
BMI categories at 20 years old	16,077	
Underweight (<18.5 kg/m^2)		1,568 (9.8)
Normal weight (18.5-24.9 kg/m^2)		13,128(81.7)
Overweight (25.0-29.9 kg/m^2)		1,176 (7.3)
Obese ($\geq$ 30.0kg/m^2)		205 (1.3)
Skirt sizes	16,933	
<=10		1,036 (6.1)
12		3,139 (18.5)
14		4,806 (28.4)
16		4,144 (24.5)
18		2,238 (13.2)
>=20		1,570 (9.3)
Blouse sizes	16,248	
<=10		1,338 (8.2)
12		3,668 (22.6)
14		4,637 (28.5)
16		3,544 (21.8)
18		1,726 (10.6)
>=20		1,335 (8.2)

For peer review only

Table 2: Incidence rates of post-menopausal breast cancer by BMI groups and groups of weight change

	n	Person-years	Cases	Incidence rate (per 1,000)
**BMI groups at enrolment**				
Underweight	327	5,051	8	1.58
Normal weight	9,813	158,418	475	2.99
Overweight	4,936	77,987	300	3.85
Obese	1,950	30,177	120	3.98
Total	17,026	271, 634	903	3.32
**BMI groups at 20 years**				
Underweight	1,568	25,006	78	3.11
Normal weight	13,125	209,950	717	3.42
Overweight	1,176	18,712	53	2.83
Obese	205	3,165	10	3.16
Total	16,074	256,834	858	3.34
**Weight change**				
Weight loss ≥5kg	988	15,536	27	1.74
Weight loss 2 - 4.9kg	745	12,116	25	2.06
Stable weight (±1.9kg)	1820	29,436	87	2.96
Weight gain 2 - 9.9kg	6287	101,530	336	3.31
Weight gain 10 - 19.9kg	4450	70,498	264	3.74
Weight gain ≥ 20kg	1969	30,632	125	4.08
Total	16,259	259,752	864	3.33

For peer review only

Table 3: Crude and adjusted hazard ratio (HR) of general and central adiposity indicators

	**Age-adjusted HR (95% CI)**	**Model 1 HR (95% CI)**	**Model 2 HR (95% CI)**
**BMI groups at 20 years**			
Cases/total	857/16,055	457/8,825	NA
Underweight	0.93 (0.73,1.15)	0.97 (0.71, 1.34)	-
Normal weight	1.00	1.00	
Overweight	0.83 (0.63,1.01)	0.81 (0.55, 1.21)	-
Obese	0.94 (0.51,1.76)	0.42 (0.10, 1.68)	-
Linear model (per kg/m^2)	0.98 (0.96, 1.00)	0.98 (0.94, 1.01)	**-**
p for trend (Linear model)	0.123	0.168	**-**
**BMI groups at enrolment**			
Cases/total	901/17,003	476/9,241	453/8,781
Underweight	0.54(0.27, 1.09)	0.49(0.16, 1.55)	0.32 (0.08,1.30)
Normal weight	1.00	1.00	1.00
Overweight	1.27(1.10, 1.47)	1.28 (1.05, 1.56)	1.34 (1.09, 1.65)
Obese	1.32 (1.08, 1.62)	1.18(0.88, 1.59)	1.27(0.91, 1.76)
Linear model (per kg/m^2)	1.03 (1.02,1.04)	1.03 (1.00,1.05)	1.04(1.02,1.07)
p for trend (Linear model)	<0.001	0.021	0.001
**Waist circumference at enrolment**			
Case/total	776/14,476	483/9,334	380/7,377
Waist circumference (per 5 cm)	1.05 (1.02,1.09)	1.06(1.01, 1.11)	1.07 (1.01,1.14)

Model 1 - Adjusted for age, education levels, parity, age at first birth, age of menarche, use of HRT, use of OCP, family history of breast cancer, dietary pattern, walking
Model 2 – Model 1 + BMI at 20 years

For peer review only

Table 3: Crude and adjusted hazard ratio (HR) of general and central adiposity indicators (continued..)

	Age-adjusted HR (95% CI)	Model 1 HR (95% CI)	Model 2 HR (95% CI)
**Skirt size at enrolment**			
Case/total	911/16,906	479/9,197	447/8,552
<10	1.00	1.00	1.00
12	1.95 (1.29,2.96)	2.06(1.12,3.78)	1.92 (1.04,3.54)
14	2.02 (1.34,3.03)	2.43(1.35,4.39)	2.45 (1.35,4.43)
16	2.31 (1.54,3.48)	2.74(1.51,4.97)	2.87 (1.58,5.22)
18	2.32 (1.52,3.55)	2.67 (1.43,4.98)	2.74 (1.45.5.15)
>20	2.57 (1.66,3.97)	3.21 (1.70,6.08)	3.06 (1.58,5.93)
Linear model (per 2 unit)	1.09(1.05,1.15)	1.12(1.06, 1.19)	1.14 (1.06,1.22)
p for trend (Linear model)	<0.001	<0.001	<0.001
**Blouse size at enrolment**			
Case/total	873/16,222	459/8,834	428/8,206
<10	1.00	1.00	1.00
12	1.56 (1.13,2.17)	2.04 (1.24, 3.36)	2.11 (1.26,3.53)
14	1.59 (1.16,2.20)	2.13 (1.30, 3.48)	2.25 (1.36,3.75)
16	1.74 (1.25,2.42)	2.20 (1.33, 3.64)	2.39 (1.42,4.03)
18	1.57 (1.09,2.26)	2.02 (1.16, 3.52)	2.21 (1.24,3.96)
>20	2.11 (1.46,3.03)	2.63 (1.50, 4.61)	2.92 (1.60,5.32)
Linear model (per 2 unit)	1.08(1.03,1.12)	1.08(1.01,1.14)	1.10(1.03,1.18)
p for trend (Linear model)	0.001	0.019	0.005

Model 1 - Adjusted for age, education levels, parity, age at first birth, age of menarche, use of HRT, use of OCP, family history of breast cancer, dietary pattern, walking

Model 2 – Model 1 + BMI at 20 years

For peer review only

Table 4: Crude and adjusted Hazard Ratio (HR) of weight change from age 20 years

	**Age-adjusted HR (95% CI)**	**Model 1 HR (95% CI)**	**Model 2 HR (95% CI)**
**Weight change**			
Cases/total	836/16,239	458/8921	453/8781
Weight loss			
≥ 5kg	0.59 (0.38,0.90)	0.64(0.34,1.19)	0.59(0.31,1.13)
2 - 4.9kg	0.69 (0.44,1.08)	0.67(0.34,1.30)	0.67(0.34,1.29)
Stable weight			
± 1.9kg	1.00	1.00	1.00
Weight gain			
2 - 9.9kg	1.12 (0.88,1.41)	1.23 (0.88,1.72)	1.23(0.88,1.72)
10-19.9kg	1.25 (0.98,1.59)	1.40 (0.99,1.98)	1.41(0.99,1.99)
≥ 20kg	1.37 (1.04, 1.80)	1.30 (0.87,1.94)	1.27(0.85,1.91)
Linear model (per kg)	1.02 (1.01, 1.02)	1.02(1.01,1.03)	1.02(1.01, 1.03)
p for trend (linear model)	<0.001	<0.001	<0.001

Model 1 - Adjusted for age, education levels, parity, age at first birth, age of menarche, use of HRT, use of OCP, family history of breast cancer, dietary pattern, walking
Model 2 – Model 1 + BMI at 20 years old

For peer review only

STROBE Statement—Checklist of items that should be included in reports of ***cohort studies***

	Item No	Recommendation	Page No.
**Title and abstract**	1	(*a*) Indicate the study's design with a commonly used term in the title or the abstract	1
		(*b*) Provide in the abstract an informative and balanced summary of what was done and what was found	1,2
**Introduction**			
Background/rationale	2	Explain the scientific background and rationale for the investigation being reported	3
Objectives	3	State specific objectives, including any prespecified hypotheses	3
**Methods**			
Study design	4	Present key elements of study design early in the paper	4
Setting	5	Describe the setting, locations, and relevant dates, including periods of recruitment, exposure, follow-up, and data collection	4
Participants	6	(*a*) Give the eligibility criteria, and the sources and methods of selection of participants. Describe methods of follow-up	4
		(*b*) For matched studies, give matching criteria and number of exposed and unexposed	
Variables	7	Clearly define all outcomes, exposures, predictors, potential confounders, and effect modifiers. Give diagnostic criteria, if applicable	4
Data sources/ measurement	8*	For each variable of interest, give sources of data and details of methods of assessment (measurement). Describe comparability of assessment methods if there is more than one group	4
Bias	9	Describe any efforts to address potential sources of bias	4
Study size	10	Explain how the study size was arrived at	4
Quantitative variables	11	Explain how quantitative variables were handled in the analyses. If applicable, describe which groupings were chosen and why	5
Statistical methods	12	(*a*) Describe all statistical methods, including those used to control for confounding	5
		(*b*) Describe any methods used to examine subgroups and interactions	5
		(*c*) Explain how missing data were addressed	5
		(*d*) If applicable, explain how loss to follow-up was addressed	5
		(*e*) Describe any sensitivity analyses	
**Results**			
Participants	13*	(a) Report numbers of individuals at each stage of study—eg numbers potentially eligible, examined for eligibility,	5

For peer review only - http://bmjopen.bmj.com/site/about/guidelines.xhtml

		confirmed eligible, included in the study, completing follow-up, and analysed	
		(b) Give reasons for non-participation at each stage	
		(c) Consider use of a flow diagram	
Descriptive data	14*	(a) Give characteristics of study participants (eg demographic, clinical, social) and information on exposures and potential confounders	5
		(b) Indicate number of participants with missing data for each variable of interest	5,6
		(c) Summarise follow-up time (eg, average and total amount)	5
Outcome data	15*	Report numbers of outcome events or summary measures over time	5.6
Main results	16	(*a*) Give unadjusted estimates and, if applicable, confounder-adjusted estimates and their precision (eg, 95% confidence interval). Make clear which confounders were adjusted for and why they were included	5,6
		(*b*) Report category boundaries when continuous variables were categorized	4
		(*c*) If relevant, consider translating estimates of relative risk into absolute risk for a meaningful time period	
Other analyses	17	Report other analyses done—eg analyses of subgroups and interactions, and sensitivity analyses	
**Discussion**			
Key results	18	Summarise key results with reference to study objectives	7
Limitations	19	Discuss limitations of the study, taking into account sources of potential bias or imprecision. Discuss both direction and magnitude of any potential bias	8
Interpretation	20	Give a cautious overall interpretation of results considering objectives, limitations, multiplicity of analyses, results from similar studies, and other relevant evidence	7,8
Generalisability	21	Discuss the generalisability (external validity) of the study results	8
**Other information**			
Funding	22	Give the source of funding and the role of the funders for the present study and, if applicable, for the original study on which the present article is based	8

*Give information separately for exposed and unexposed groups.

Note: An Explanation and Elaboration article discusses each checklist item and gives methodological background and published examples of transparent reporting. The STROBE checklist is best used in conjunction with this article (freely available on the Web sites of PLoS Medicine at http://www.plosmedicine.org/, Annals of Internal Medicine at http://www.annals.org/, and Epidemiology at http://www.epidem.com/). Information on the STROBE Initiative is available at http://www.strobe-statement.org.

For peer review only - http://bmjopen.bmj.com/site/about/guidelines.xhtml

Associations of clothing size, adiposity and weight change with risk of post-menopausal breast cancer in the UK Women's Cohort Study (UKWCS)

Journal:	*BMJ Open*
Manuscript ID	bmjopen-2018-022599.R1
Article Type:	Research
Date Submitted by the Author:	24-Jul-2018
Complete List of Authors:	Moy, Foong Ming; University of Malaya, Social & Preventive Medicine, Faculty of Medicine<br>Greenwood, Darren; University of Leeds, Division of Epidemiology & Biostatistics, School of Medicine<br>Cade, Janet; University of Leeds, Nutritional Epidemiology Group, School of Food Science and Nutrition
**Primary Subject Heading**:	Public health
Secondary Subject Heading:	Epidemiology
Keywords:	blouse size, skirt size, weight change, post-menopausal breast cancer, UK Women Cohort

SCHOLARONE™
Manuscripts

For peer review only - http://bmjopen.bmj.com/site/about/guidelines.xhtml

Associations of clothing size, adiposity and weight change with risk of post-menopausal breast cancer in the UK Women's Cohort Study (UKWCS)

Foong Ming Moy [1,2], Darren C Greenwood[3], Janet E Cade[2]

[1]Julius Centre University of Malaya, Department of Social & Preventive Medicine, Faculty of Medicine, University of Malaya, 50603 Kuala Lumpur, Malaysia

[2]Nutritional Epidemiology Group, School of Food Science & Nutrition, University of Leeds, Leeds, LS2 9JT, United Kingdom

[3] Division of Epidemiology & Biostatistics, School of Medicine, University of Leeds, Leeds, LS2 9NL, United Kingdom

Corresponding author:

Dr Foong Ming Moy,

Julius Centre University of Malaya, Department of Social & Preventive Medicine, Faculty of Medicine, University of Malaya, Kuala Lumpur, Malaysia

Email: moyfm@ummc.edu.my

For peer review only

For peer review only - http://bmjopen.bmj.com/site/about/guidelines.xhtml

Abstract:

Objectives: Breast cancer is associated with overweight and obesity after menopause. However, clothing size as a proxy of adiposity in predicting post-menopausal breast cancer is not widely studied. We aimed to explore the relationships between post-menopausal breast cancer risk with adipose indicators (including clothing sizes) and weight change over adulthood.

Design: Prospective cohort study

Setting: England, Wales and Scotland

Participants: 17,781 post-menopausal women from the UK Women's Cohort Study

Primary outcome measure: Incident cases of malignant breast cancers (ICD9 Code 174 and ICD 10 Code C50)

Results: From 282,277 person years follow up, there were 946 incident breast cancer cases with an incidence rate of 3.35 per 1,000 women. BMI (HR: 1.04; 95% CI: 1.02, 1.07), blouse size (HR: 1.10; 1.03, 1.18), waist circumference (HR: 1.07; 1.01, 1.14) and skirt size (HR: 1.14 (1.06, 1.22) had positive associations with post-menopausal breast cancer after adjustment for potential confounders. Increased weight over adulthood (HR: 1.02; 1.01, 1.03) was also associated with increased risk for post-menopausal breast cancer.

Conclusions: Blouse and skirt sizes can be used as adipose indicators in predicting post-menopausal breast cancer. Maintaining healthy body weight over adulthood is an effective measure in the prevention of post-menopausal breast cancer.

Strengths and limitations of this study:

This study may be the first to report the use of both blouse and skirt sizes as measures of body size.

The large study sample with long follow up duration and linkage of participants' ID with the National Statistics National Health Service central register provides accurate information on breast cancer diagnosis.

All adipose indicators, blouse and skirt sizes were self-reported.

We were unable to evaluate in more detail the effect of weight gain at different ages.

Breast tumour characteristics were not available and we could not explore whether our findings may differ by breast cancer subtypes.

Keywords: blouse size, skirt size, adiposity, weight change, post-menopausal breast cancer, UK Women Cohort

For peer review only

For peer review only - http://bmjopen.bmj.com/site/about/guidelines.xhtml

Introduction:

Breast cancer is the most common cancer among women worldwide, accounting for 25% of new cases.[1] In the UK, it is also the most common cancer among women with around 54,800 cases diagnosed in 2014. [2] The prevalence of obesity in the UK has increased from 15% in 1993 to 27% in 2015, and 57% of the women were overweight or obese.[3] Obesity is associated with increased risks of chronic diseases and certain cancers.[4,5] There is strong evidence that obesity and weight gain over adult life are associated with increased risk of post-menopausal breast cancer.[6,7] Maintaining healthy weight is a key recommendation to prevent post-menopausal breast cancer.[6]

To date, many adipose indicators namely weight, body mass index (BMI), waist circumference, waist to hip ratio are used in research and health education programmes. Body Mass Index (BMI) is an established indicator for general obesity, while central obesity is commonly measured using waist circumference. Among post-menopausal women, higher BMI and larger waist circumference during adulthood; and weight gain over adulthood are associated with increased breast cancer risk.[6]

Clothing sizes are objective, easy to recall and less likely to be misreported. From a public health perspective, clothing sizes may be useful when anthropometric measurements are not available or challenging in their measurements due to extreme obesity. Skirt or trouser size[8-10] have been suggested to be used as proxy for central obesity. Skirt size appears to predict cancer risk independently of BMI.[10] Change in skirt size was also found to be predictive of post-menopausal breast cancer.[11] However, blouse size has not been explored as a proxy for general obesity in predicting post-menopausal breast cancer. In view of the limited evidence of using clothing size as an adiposity indicator in predicting post-menopausal breast cancer risk, and the ease of self-reporting of these measures, we have used data from the UK Women's Cohort Study (UKWCS) to explore the relationships between post-menopausal breast cancer risk with various adipose indicators (including skirt and blouse sizes); as well as weight change over adulthood.

Materials and methods:

Study design and population

The recruitment of participants and characteristics of the UKWCS have been described in detail previously.[12] In brief, a total of 35 792 women were recruited between 1995 and 1998 via a postal questionnaire. They were aged 35 to 69 years at baseline and were living in England, Wales and Scotland. In total, 17 781 women were post-menopausal and 15 951 women were pre-menopausal at baseline. Post-menopausal women were those who had not had a natural menstrual period in the last 12 months, or were older than 50 years old and currently on hormone replacement therapy (HRT), or with a previous hysterectomy and HRT. Where these data were missing, if the woman was aged more than 50 years she was also counted as menopausal. Only post-menopausal women were included in the current analyses.

For peer review only

Ethics clearance

A total of 174 local research ethics committees were contacted and permission to carry out the baseline UKWCS study was obtained.[13] Participants had consented to the use of information gathered at baseline, future phases and cancer registries for research purposes provided that confidentiality was maintained. The National Research Ethics Committee for Yorkshire and the Humber, Leeds East have now taken on responsibility for the on-going cohort.[14]

Patient and Public Involvement

The participants were not involved in the development of the research question and outcome measures. They were also not involved in the design and conduct of the study. The results are not disseminated to the participants apart from the UKWCS website.

Case definition and ascertainment

All women were flagged for death and cancer registration on the office of National Statistics National Health Service central register. Causes of death and cancer diagnosis were coded in the International Classification of Diseases (ICD) 9 and 10. All malignant breast cancers (ICD9 Code 174 and ICD 10 Code C50) registered after the participants returned their questionnaires were taken as incident cases. Prevalent cancers identified from the questionnaires and incident cases identified within one year from the date of questionnaire returned were excluded to rule out reverse causation since undiagnosed breast cancer may influence weight.

Assessment of adipose indicators

Anthropometric measures such as weight, height, waist and hip circumference at enrolment; and weight at age 20 years were self-recorded. Body mass index (BMI) was categorised into groups of underweight, normal weight, overweight and obese according to the WHO criteria.[15] Weight change over adulthood was computed by weight at enrolment minus weight at 20 years old. Weight change was further categorised into stable weight ($\pm$ 2kg), weight loss (2 to 4.9kg and $\geq$ 5kg) and weight gain (2 to 9.9kg, 10 to 19.9kg and $\geq$20kg). Skirt size and blouse size at enrolment were also reported. The clothing sizes ranged from 6 to 26 with the increase in units of 2.

Other confounding factors

Socio-economic characteristics, dietary pattern (meat eaters, fish eaters or vegetarian), lifestyle (smoking, alcohol consumption, physical activity etc), reproductive history, information on past health experience, sibling and parental health were reported at baseline.

Statistical analysis

The follow up (person-years) for each participant was counted from the beginning of the study until the date of breast cancer diagnosis or the censor date (1 April 2014), whichever occurred first. The median

For peer review only - http://bmjopen.bmj.com/site/about/guidelines.xhtml

follow up was 16.9 years. The relationships between adipose indicators and weight change; and post-menopausal breast cancer were explored using Cox's proportional hazards regression. Hazard ratios with 95% confidence interval were reported. Linear trends, using the exposure categories as a continuous variable were also presented. Potential confounders such as age, highest education levels achieved, time spent walking (hours/day), family history of breast cancer, oral contraceptive (OC) use, hormone replacement therapy (HRT) use, parity, age at first birth, age of menarche, and dietary pattern (meat eaters, fish eaters and vegetarians) were included in the fully adjusted models. In addition, BMI at 20 years was also adjusted for all adipose indicators, except BMI at 20 years. All statistical analyses were conducted using Stata version 14.

Results

After excluding the prevalent cases at baseline and incident cases developed within one year of questionnaire returned, 17,715 post-menopausal women were included in this analysis. From 282,277 person years follow up, there were 946 incident breast cancer cases with an incidence rate of 3.35 per 1,000 women.

The mean (SD) age of the post-menopausal participants at enrolment was 58.8 (7.5) years. The majority were white (99.1%); about one fifth had tertiary qualifications; 10% were smokers; 11% self-reported being on a vegetarian diet; 3% reported a family history of breast cancer; about 41% had ever taken HRT; 52% ever used OCP and 17% were nulliparous. The mean (SD) BMI at enrolment and at 20 years of age were 24.9(4.3) kg/m^2 and 21.5(2.9) kg/m^2 respectively (Table 1). The correlation between skirt size and blouse size was very high (r=0.91, p<0.001) while the correlation between BMI and waist circumference was only moderate (r=0.65, p<0.001). Skirt size was highly correlated with waist circumference (r=0.76, p<0.0001) while blouse size was highly correlated with BMI (0.78, p<0.0001) (Supplementary table).

For peer review only - http://bmjopen.bmj.com/site/about/guidelines.xhtml

141 Table 1: Descriptive characteristics of post-menopausal women

	Total	n (%)
Age (mean + SD) years	17,686	58.8+7.5
Highest education achieved	15,521	
No education		3,950 (25.5)
O-level		4,682 (30.2)
A-level		3,700 (23.8)
Degree		3,189 (20.6)
White	17,149	16, 995 (99.1)
Food groups	15,602	
Meat eaters		12,175 (78.0)
Fish eaters		1,700 (10.9)
Vegetarians		1,727 (11.1)
Smokers	17,714	1,758 (9.9)
Walking (mean + SD) (hours /day)	15,866	0.94+0.77
Family history of breast cancer	17,715	539 (3.0)
Ever taken HRT	17,031	7,045 (41.4)
Ever used OC	17,362	8,997 (51.8)
Parity	17,292	
0		2,937 (16.9)
1-2		8,685 (50.2)
3-4		5,129 (29.7)
>=5		541 (3.1)
Age of menarche (mean + SD) (years)	17,247	12.9+1.6
Age of first birth (mean + SD) (years)	14,336	25.3+4.4
BMI at enrolment (mean + SD) (kg/m^2)	17,029	24.9+4.3
Waist circumference (mean + SD) (cm)	14,491	75.3+9.7
BMI at 20 years (mean + SD) (kg/m^2)	16,077	21.5+2.9
Weight at 20 years (mean + SD) (kg)	16,388	57.5+8.3

For peer review only - http://bmjopen.bmj.com/site/about/guidelines.xhtml

Table 1: Descriptive characteristics of post-menopausal women (n=17,715) (Continued..)

BMI categories at enrolment	17,029	
Underweight (<18.5 kg/m^2)		327 (1.9)
Normal weight (18.5-24.9 kg/m^2)		9,816 (57.6)
Overweight (25.0-29.9 kg/m^2)		4,936 (29.0)
Obese ($\geq$ 30.0kg/m^2)		1,950 (11. 5)
BMI categories at 20 years old	16,077	
Underweight (<18.5 kg/m^2)		1,568 (9.8)
Normal weight (18.5-24.9 kg/m^2)		13,128(81.7)
Overweight (25.0-29.9 kg/m^2)		1,176 (7.3)
Obese ($\geq$ 30.0kg/m^2)		205 (1.3)
Skirt sizes	16,933	
<=10		1,036 (6.1)
12		3,139 (18.5)
14		4,806 (28.4)
16		4,144 (24.5)
18		2,238 (13.2)
>=20		1,570 (9.3)
Blouse sizes	16,248	
<=10		1,338 (8.2)
12		3,668 (22.6)
14		4,637 (28.5)
16		3,544 (21.8)
18		1,726 (10.6)
>=20		1,335 (8.2)

For peer review only

For peer review only - http://bmjopen.bmj.com/site/about/guidelines.xhtml

145 Incidence rates (per 1,000 women) for post-menopausal breast cancer increased by BMI categories at enrolment, BMI categories at 20 years old and weight change (from 20 years old to enrolment age) (Table 2). Obese women at recruitment had the highest incidence rates while women with the highest weight loss from age 20 had the lowest incidence rate of post-menopausal breast cancer.

Table 2: Incidence rates of post-menopausal breast cancer by BMI groups and groups of weight change

	n	Person-years	Cases	Incidence rate (per 1,000)
**BMI groups at enrolment**				
Underweight	327	5,051	8	1.58
Normal weight	9,813	158,418	475	2.99
Overweight	4,936	77,987	300	3.85
Obese	1,950	30,177	120	3.98

149

150

For peer review only - http://bmjopen.bmj.com/site/about/guidelines.xhtml

Total	17,026	271, 634	903	3.32
**BMI groups at 20 years**				
Underweight	1,568	25,006	78	3.11
Normal weight	13,125	209,950	717	3.42
Overweight	1,176	18,712	53	2.83
Obese	205	3,165	10	3.16
Total	16,074	256,834	858	3.34
**Weight change**				
Weight loss ≥5kg	988	15,536	27	1.74
Weight loss 2 - 4.9kg	745	12,116	25	2.06
Stable weight (±1.9kg)	1820	29,436	87	2.96
Weight gain 2 - 9.9kg	6287	101,530	336	3.31
Weight gain 10 - 19.9kg	4450	70,498	264	3.74
Weight gain ≥ 20kg	1969	30,632	125	4.08
Total	16,259	259,752	864	3.33

For peer review only

After adjustment for potential confounders, adipose indicators at enrolment namely BMI, waist circumference and clothing sizes (blouse and skirt sizes) were associated with increased risk for post-menopausal breast cancer (Table 3). Post-menopausal women who were overweight (HR: 1.34, 95% CI: 1.09, 1.65) or obese (HR: 1.27, 95% CI: 0.91, 1.76) had higher risks of breast cancer compared to those with normal weight at enrolment. There was no association observed for women in the underweight category. No association was observed for BMI at 20 years with post-menopausal breast cancer risk.

Table 3: Crude and adjusted hazard ratio (HR) of general and central adiposity indicators

	Age-adjusted HR (95% CI)	Model 1 HR (95% CI)	Model 2 HR (95% CI)
**BMI groups at 20 years**			
Cases/total	857/16,055	457/8,825	NA
Underweight	0.93 (0.73,1.15)	0.97 (0.71, 1.34)	-
Normal weight	1.00	1.00	
Overweight	0.83 (0.63,1.01)	0.81 (0.55, 1.21)	-
Obese	0.94 (0.51,1.76)	0.42 (0.10, 1.68)	-
Linear model (per kg/m^2)	0.98 (0.96, 1.00)	0.98 (0.94, 1.01)	-
p for trend (Linear model)	0.123	0.168	-
**BMI groups at enrolment**			
Cases/total	901/17,003	476/9,241	453/8,781
Underweight	0.54(0.27, 1.09)	0.49(0.16, 1.55)	0.32 (0.08,1.30)
Normal weight	1.00	1.00	1.00
Overweight	1.27(1.10, 1.47)	1.28 (1.05, 1.56)	1.34 (1.09, 1.65)
Obese	1.32 (1.08, 1.62)	1.18(0.88, 1.59)	1.27(0.91, 1.76)
Linear model (per kg/m^2)	1.03 (1.02,1.04)	1.03 (1.00,1.05)	1.04(1.02,1.07)
p for trend (Linear model)	<0.001	0.021	0.001
**Waist circumference at enrolment**			
Case/total	776/14,476	483/9,334	380/7,377
Waist circumference (per 5 cm)	1.05 (1.02,1.09)	1.06(1.01, 1.11)	1.07 (1.01,1.14)

Model 1 - Adjusted for age, education levels, parity, age at first birth, age of menarche, use of HRT, use of OC, family history of breast cancer, dietary pattern, walking (hours/day)
Model 2 – Model 1 + BMI at 20 years

For peer review only - http://bmjopen.bmj.com/site/about/guidelines.xhtml

Table 3: Crude and adjusted hazard ratio (HR) of general and central adiposity indicators (continued..)

	Age-adjusted HR (95% CI)	Model 1 HR (95% CI)	Model 2 HR (95% CI)
**Skirt size at enrolment**			
Case/total	911/16,906	479/9,197	447/8,552
<10	1.00	1.00	1.00
12	1.95 (1.29,2.96)	2.06(1.12,3.78)	1.92 (1.04,3.54)
14	2.02 (1.34,3.03)	2.43(1.35,4.39)	2.45 (1.35,4.43)
16	2.31 (1.54,3.48)	2.74(1.51,4.97)	2.87 (1.58,5.22)
18	2.32 (1.52,3.55)	2.67 (1.43,4.98)	2.74 (1.45.5.15)
>20	2.57 (1.66,3.97)	3.21 (1.70,6.08)	3.06 (1.58,5.93)
Linear model (per 2 unit)	1.09(1.05,1.15)	1.12(1.06, 1.19)	1.14 (1.06,1.22)
p for trend (Linear model)	<0.001	<0.001	<0.001
**Blouse size at enrolment**			
Case/total	873/16,222	459/8,834	428/8,206
<10	1.00	1.00	1.00
12	1.56 (1.13,2.17)	2.04 (1.24, 3.36)	2.11 (1.26,3.53)
14	1.59 (1.16,2.20)	2.13 (1.30, 3.48)	2.25 (1.36,3.75)
16	1.74 (1.25,2.42)	2.20 (1.33, 3.64)	2.39 (1.42,4.03)
18	1.57 (1.09,2.26)	2.02 (1.16, 3.52)	2.21 (1.24,3.96)
>20	2.11 (1.46,3.03)	2.63 (1.50, 4.61)	2.92 (1.60,5.32)
Linear model (per 2 unit)	1.08(1.03,1.12)	1.08(1.01,1.14)	1.10(1.03,1.18)
p for trend (Linear model)	0.001	0.019	0.005

164 Model 1 - Adjusted for age, education levels, parity, age at first birth, age of menarche, use of HRT, use
165 of OC, family history of breast cancer, dietary pattern, walking (hours/day)
166 Model 2 – Model 1 + BMI at 20 years
167

For peer review only - http://bmjopen.bmj.com/site/about/guidelines.xhtml

BMI and blouse size (which indicate general obesity) as well as waist circumference and skirt size (which indicate central obesity) had positive association with post-menopausal breast cancer. There was an increase of 3% in HR for every unit increase in BMI and 6% in HR for every 5cm increase in waist circumference after adjustment for confounders. There was an additional 1% increase in HRs after additionally adjusted for BMI at 20 years for both BMI and waist circumference. For every 2-unit increase in blouse and skirt sizes (as UK clothes size increased by 2 units), the HRs for blouse and skirt sizes were 1.08 (95% CI: 1.01, 1.14) and 1.12 (95% CI: 1.06, 1.19) respectively. The HRs increased to 1.10 (95% CI: 1.03, 1.18) and 1.14 (95% CI: 1.06, 1.22) for blouse and skirt sizes respectively after additional adjustment for BMI at 20 years of age.

Weight change over adulthood was associated with increased risk for post-menopausal breast cancer (HR: 1.02, 95% CI: 1.01, 1.03). For every 1kg weight change, there was a corresponding 2% risk increase after adjusting for confounders, similar results were observed when weight change was additionally adjusted for BMI at 20 years (Table 4). There was an increasing trend in associated risks with post-menopausal breast cancer (P for trend <0.001) when weight change was categorised into groups of weight loss, stable weight and increased weight, using stable weight as the reference category.

For peer review only - http://bmjopen.bmj.com/site/about/guidelines.xhtml

Table 4: Crude and adjusted Hazard Ratio (HR) of weight change from age 20 years

	Age-adjusted HR (95% CI)	Model 1 HR (95% CI)	Model 2 HR (95% CI)
**Weight change**			
Cases/total	836/16,239	458/8921	453/8781
Weight loss			
≥ 5kg	0.59 (0.38,0.90)	0.64(0.34,1.19)	0.59(0.31,1.13)
2 - 4.9kg	0.69 (0.44,1.08)	0.67(0.34,1.30)	0.67(0.34,1.29)
Stable weight			
± 1.9kg	1.00	1.00	1.00
Weight gain			
2 - 9.9kg	1.12 (0.88,1.41)	1.23 (0.88,1.72)	1.23(0.88,1.72)
10-19.9kg	1.25 (0.98,1.59)	1.40 (0.99,1.98)	1.41(0.99,1.99)
≥ 20kg	1.37 (1.04, 1.80)	1.30 (0.87,1.94)	1.27(0.85,1.91)
Linear model (per kg)	1.02 (1.01, 1.02)	1.02(1.01,1.03)	1.02(1.01, 1.03)
p for trend (linear model)	<0.001	<0.001	<0.001

Model 1 - Adjusted for age, education levels, parity, age at first birth, age of menarche, use of HRT, use of OC, family history of breast cancer, dietary pattern, walking (hours/day)
Model 2 – Model 1 + BMI at 20 years old

Discussion

Our results suggested that clothing size such as blouse and skirt sizes can be used as adipose indicators in predicting post-menopausal breast cancer. Trouser and skirt size have been reported to predict cancer risks independent of BMI,[10] while skirt size changes between 20 years of age and postmenopausal-age was reported to be associated with post-menopausal breast cancer.[11] However, blouse size has not been explored as a proxy for general obesity in predicting post-menopausal breast cancer. Our study confirms and provides additional evidence that skirt size is associated with the risk of postmenopausal breast cancer. In addition, our study may be the first to provide evidence that larger blouse size is associated with increased risk of post-menopausal breast cancer. The clothing sizes will provide additional indicators as proxy for adiposity if patients fail to recall (especially the elderly) or feel embarrassed to disclose their weight or BMI (for those who are obese).

Skirt size and waist circumference were highly correlated and performed equally well in predicting post-menopausal breast cancer risk. Our findings provide additional evidence of using skirt size as a proxy for central obesity in predicting post-menopausal breast cancer. On the other hand, there was no previous

For peer review only

For peer review only - http://bmjopen.bmj.com/site/about/guidelines.xhtml

report on blouse size associated with increased risk of post-menopausal breast cancer. Blouse size could serve as an indicator for general obesity as it has a strong correlation with BMI. Blouse size also performed as well as BMI in predicting breast cancer among post-menopausal women. A previous study conducted in the UK reported that among 362 respondents, only 3% reported a clothing size different from their clothing labels.[9] This indicated that self-reported clothing size is reliable. Blouse and skirt sizes which are simple and non-invasive self-reported measures could be promoted as proxies for adipose indictors. From a public health perspective, these findings are significant when anthropometric measurements are not available, or challenging in their measurements due to extreme obesity.[10]

Overweight and obese women at enrolment had up to 32% increased risk of post-menopausal breast cancer. The association between overweight / obesity with post-menopausal breast cancer remained after further adjustment for BMI at 20 years. Women who gained the most weight since the age of 20 years old, had almost 30% increased risk of post-menopausal breast cancer after adjustment for potential confounders and BMI at 20 years. Similar findings have been reported by other researchers.[16-19]

Different periods of weight gain (ie: between age 18 years and the current age, between ages 18 and 35 years, between ages 35 and 50 years, and between age 50 years and the current age) were consistently associated with increased breast cancer risk.[20] Similar findings were reported by Eliassen et al[21] that women who gained weight since age 18 years or since menopause were at an increased risk of breast cancer. These results suggest that regardless of BMI in early adulthood, being obese in later life or gaining weight along the way increases deposition of adipose tissue. After menopause, adipose tissue is the major location for the synthesis of oestrogens. Circulating oestrogen are higher in obese than in slim post-menopausal women.[19] Obesity also contributes to inflammatory changes leading to macrophage polarization and altered adipokine profile.[22] These mechanisms may explain the association which links obesity and post-menopausal breast cancer together.

Consistent with previous literature;[6,11,23] waist circumference as an indicator for central obesity was found to be positively associated with post-menopausal breast cancer. The association remained after adjustment for potential confounders and BMI at 20 years. This may indicate that regardless of being obese in early adulthood or at later life, being centrally obese in later life increases the risk of post-menopausal breast cancers. Central obesity is associated with increased insulin levels and insulin like growth factors which may stimulate the growth of breast cancer cells and neoplastic degeneration.[24]

According to the latest report (2017) from the World Cancer Research Fund International / American Institute for Cancer Research, being overweight or obese during young adulthood decreases the risk of post-menopausal breast cancer.[6] Low levels of adiposity in the mammary gland may alter breast tissue maturation, making breast tissue more susceptible to carcinogenic stimuli among leaner women. Sex hormone levels may also explain the inverse relationship between early life adiposity and breast cancer risk. However, we did not find BMI at 20 years to be negatively associated with post-menopausal breast cancer incidence, as also reported by other cohort studies.[16,25]

For peer review only - http://bmjopen.bmj.com/site/about/guidelines.xhtml

There are a few limitations which need to be considered while interpreting the results. All adipose indicators, blouse and skirt sizes were self-reported. Some overweight or obese women may be more likely to under-report their weight compared to normal weight participants. On the other hand, blouse and skirt sizes offer an objective standard as a proxy to report overweight / obesity, which are potentially less liable for misreporting. We were unable to evaluate in more detail the effect of weight gain at different ages, other than that of weight at age 20 years and weight at enrolment. Breast tumour characteristics were not available and we could not explore whether our findings may differ by breast cancer subtypes. On the other hand, we believe we are the first to report that both blouse and skirt sizes predict post-menopausal breast cancer risk equally as well as other more invasive measures of body size. In addition, our study sample is large with long follow up duration. The linkage of participants' ID with the National Statistics National Health Service central register provides accurate information on breast cancer diagnosis.

Conclusion

Overweight/obesity (BMI and blouse size), central obesity (waist circumference and skirt size) and weight change during adulthood are associated with increased risk of post-menopausal breast cancer. Clothing size such as skirt and blouse sizes can be used as a proxy for adipose indicators in predicting post-menopausal breast cancer. Clothing sizes offer an objective standard as a proxy to report overweight / obesity, which are useful in the public health setting. Maintaining healthy body weight over adulthood is an effective measure in the prevention of post-menopausal breast cancer.

Acknowledgements:

We would like to acknowledge all women who participated in our study. The support from the Nutritional Epidemiology Group, University of Leeds is also acknowledged.

Funding:

JC was funded by a Medical Research Council grant no: MR/L02019X/1.

Contributors:

Conception and design: FMM, JEC and DCG. Analysis and interpretation: FMM, JEC and DCG. Data collection: JEC and DCG. Manuscript preparation: FMM, JEC and DCG. Overall responsibility: FMM

Disclaimer:

The authors are responsible for the content and writing of this article. JEC had full access to all of the data in the study and takes responsibility for the integrity of the data. FMM, JEC and DCG takes responsibility of the accuracy of data analysis.

Competing interests:

None declared

For peer review only - http://bmjopen.bmj.com/site/about/guidelines.xhtml

Data sharing statement:

The UK Women's Cohort Study is available for researchers to use at the following link: https://data.cdrc.ac.uk/dataset/uk-womens-cohort-questionnaire-data. The data is safeguarded and forms to apply to access the data can be found here: https://www.cdrc.ac.uk/data-services/using-our-data/.

For peer review only

For peer review only - http://bmjopen.bmj.com/site/about/guidelines.xhtml

References:

1. IARC, WHO. GLOBOCAN 2012: Estimated cancer incidence, mortality and prevalence worldwide in 2012. Secondary GLOBOCAN 2012: Estimated cancer incidence, mortality and prevalence worldwide in 2012 2012. http://globocan.iarc.fr/Pages/fact_sheets_cancer.aspx.
2. Cancer Research UK Breast cancer statistics : Breast cancer incidence. Secondary Breast cancer statistics : Breast cancer incidence 2014. http://www.cancerresearchuk.org/health-professional/cancer-statistics/statistics-by-cancer-type/breast-cancer.
3. NHS Digital. Statistics on Obesity, Physical Activity and Diet, England 2017: Report, 2017.
4. Wang J, Yang DL, Chen ZZ, et al. Associations of body mass index with cancer incidence among populations, genders, and menopausal status: A systematic review and meta-analysis. Cancer epidemiology 2016;**42**:1-8 doi: 10.1016/j.canep.2016.02.010[published Online First: Epub Date]|.
5. Renehan AG, Soerjomataram I. Obesity as an Avoidable Cause of Cancer (Attributable Risks). Recent results in cancer research Fortschritte der Krebsforschung Progres dans les recherches sur le cancer 2016;**208**:243-56 doi: 10.1007/978-3-319-42542-9_13[published Online First: Epub Date]|.
6. World Cancer Research Fund International / American Institute for Cancer Research. Continuous Update Project Report: Diet, nutrition, physical activity and breast cancer. Secondary Continuous Update Project Report: Diet, nutrition, physical activity and breast cancer 2017. wcrf.org/breast-cancer-2017.
7. Tamimi RM, Spiegelman D, Smith-Warner SA, et al. Population Attributable Risk of Modifiable and Nonmodifiable Breast Cancer Risk Factors in Postmenopausal Breast Cancer. American journal of epidemiology 2016;**184**(12):884-93 doi: 10.1093/aje/kww145[published Online First: Epub Date]|.
8. Battram DS, Beynon C, He M. The reliability and validity of using clothing size as a proxy for waist circumference measurement in adults. Applied Physiology, Nutrition, and Metabolism 2011;**36**(2):183-90 doi: 10.1139/h11-001[published Online First: Epub Date]|.
9. Han TS, Gates E, Truscott E, et al. Clothing size as an indicator of adiposity, ischaemic heart disease and cardiovascular risks. J Hum Nutr Diet 2005;**18**(6):423-30
10. Hughes LA, Schouten LJ, Goldbohm RA, et al. Self-reported clothing size as a proxy measure for body size. Epidemiology (Cambridge, Mass) 2009;**20**(5):673-6 doi: 10.1097/EDE.0b013e3181a66eb5[published Online First: Epub Date]|.
11. Fourkala EO, Burnell M, Cox C, et al. Association of skirt size and postmenopausal breast cancer risk in older women: a cohort study within the UK Collaborative Trial of Ovarian Cancer Screening (UKCTOCS). BMJ open 2014;**4**(9):e005400 doi: 10.1136/bmjopen-2014-005400[published Online First: Epub Date]|.
12. Cade JE, Burley VJ, Alwan NA, et al. Cohort Profile: The UK Women's Cohort Study (UKWCS). International journal of epidemiology 2017;**46**(2):e11 doi: 10.1093/ije/dyv173[published Online First: Epub Date]|.
13. von Koerber K, Bader N, Leitzmann C. Wholesome Nutrition: an example for a sustainable diet. Proc Nutr Soc 2017;**76**(1):34-41 doi: 10.1017/s0029665116000616[published Online First: Epub Date]|.
14. Nelson ME, Hamm MW, Hu FB, et al. Alignment of Healthy Dietary Patterns and Environmental Sustainability: A Systematic Review. Adv Nutr 2016;**7**(6):1005-25 doi: 10.3945/an.116.012567[published Online First: Epub Date]|.
15. WHO. Preventing and managing the global epidemic. Report of a WHO Consultation on Obesity. . Geneva: World Health Organisation, 1998.

For peer review only

For peer review only - http://bmjopen.bmj.com/site/about/guidelines.xhtml

16. Han X, Stevens J, Truesdale KP, et al. Body mass index at early adulthood, subsequent weight change and cancer incidence and mortality. International journal of cancer 2014;**135**(12):2900-9 doi: 10.1002/ijc.28930[published Online First: Epub Date]|.
17. Alsaker MD, Janszky I, Opdahl S, et al. Weight change in adulthood and risk of postmenopausal breast cancer: the HUNT study of Norway. British journal of cancer 2013;**109**(5):1310-7 doi: 10.1038/bjc.2013.403[published Online First: Epub Date]|.
18. Rosner B, Eliassen AH, Toriola AT, et al. Weight and weight changes in early adulthood and later breast cancer risk. International journal of cancer 2017;**140**(9):2003-14 doi: 10.1002/ijc.30627[published Online First: Epub Date]|.
19. Welti LM, Beavers DP, Caan BJ, et al. Weight Fluctuation and Cancer Risk in Postmenopausal Women: The Women's Health Initiative. Cancer epidemiology, biomarkers & prevention : a publication of the American Association for Cancer Research, cosponsored by the American Society of Preventive Oncology 2017;**26**(5):779-86 doi: 10.1158/1055-9965.EPI-16-0611[published Online First: Epub Date]|.
20. Ahn J, Schatzkin A, Lacey JV, Jr., et al. Adiposity, adult weight change, and postmenopausal breast cancer risk. Archives of internal medicine 2007;**167**(19):2091-102 doi: 10.1001/archinte.167.19.2091[published Online First: Epub Date]|.
21. Eliassen AH, Colditz GA, Rosner B, et al. Adult weight change and risk of postmenopausal breast cancer. Jama 2006;**296**(2):193-201 doi: 10.1001/jama.296.2.193[published Online First: Epub Date]|.
22. Perez-Hernandez AI, Catalan V, Gomez-Ambrosi J, et al. Mechanisms linking excess adiposity and carcinogenesis promotion. Frontiers in endocrinology 2014;**5**:65 doi: 10.3389/fendo.2014.00065[published Online First: Epub Date]|.
23. Harvie M, Hooper L, Howell AH. Central obesity and breast cancer risk: a systematic review. Obesity reviews : an official journal of the International Association for the Study of Obesity 2003;**4**(3):157-73
24. Rose DP, Komninou D, Stephenson GD. Obesity, adipocytokines, and insulin resistance in breast cancer. Obesity reviews : an official journal of the International Association for the Study of Obesity 2004;**5**(3):153-65 doi: 10.1111/j.1467-789X.2004.00142.x[published Online First: Epub Date]|.
25. Krishnan K, Bassett JK, MacInnis RJ, et al. Associations between weight in early adulthood, change in weight, and breast cancer risk in postmenopausal women. Cancer epidemiology, biomarkers & prevention : a publication of the American Association for Cancer Research, cosponsored by the American Society of Preventive Oncology 2013;**22**(8):1409-16 doi: 10.1158/1055-9965.EPI-13-0136[published Online First: Epub Date]|.

For peer review only - http://bmjopen.bmj.com/site/about/guidelines.xhtml

Supplementary Table:

Correlations between Skirt size, blouse size, waist circumference and BMI

	Skirt size	Blouse size	Waist Circumference	BMI
Skirt size	1.00			
Blouse size	0.91	1.00		
Waist circumference	0.76	0.73	1.00	
BMI	0.75	0.78	0.65	1.00

All p values <0.001

For peer review only

STROBE Statement—Checklist of items that should be included in reports of ***cohort studies***

	Item No	Recommendation	Page No.
**Title and abstract**	1	(*a*) Indicate the study's design with a commonly used term in the title or the abstract	1
		(*b*) Provide in the abstract an informative and balanced summary of what was done and what was found	1,2
**Introduction**			
Background/rationale	2	Explain the scientific background and rationale for the investigation being reported	3
Objectives	3	State specific objectives, including any prespecified hypotheses	3
**Methods**			
Study design	4	Present key elements of study design early in the paper	4
Setting	5	Describe the setting, locations, and relevant dates, including periods of recruitment, exposure, follow-up, and data collection	4
Participants	6	(*a*) Give the eligibility criteria, and the sources and methods of selection of participants. Describe methods of follow-up	4
		(*b*) For matched studies, give matching criteria and number of exposed and unexposed	
Variables	7	Clearly define all outcomes, exposures, predictors, potential confounders, and effect modifiers. Give diagnostic criteria, if applicable	4
Data sources/ measurement	8*	For each variable of interest, give sources of data and details of methods of assessment (measurement). Describe comparability of assessment methods if there is more than one group	4
Bias	9	Describe any efforts to address potential sources of bias	4
Study size	10	Explain how the study size was arrived at	4
Quantitative variables	11	Explain how quantitative variables were handled in the analyses. If applicable, describe which groupings were chosen and why	5
Statistical methods	12	(*a*) Describe all statistical methods, including those used to control for confounding	5
		(*b*) Describe any methods used to examine subgroups and interactions	5
		(*c*) Explain how missing data were addressed	5
		(*d*) If applicable, explain how loss to follow-up was addressed	5
		(*e*) Describe any sensitivity analyses	
**Results**			
Participants	13*	(a) Report numbers of individuals at each stage of study—eg numbers potentially eligible, examined for eligibility,	5

For peer review only - http://bmjopen.bmj.com/site/about/guidelines.xhtml

For peer review only

		confirmed eligible, included in the study, completing follow-up, and analysed	
		(b) Give reasons for non-participation at each stage	
		(c) Consider use of a flow diagram	
Descriptive data	14*	(a) Give characteristics of study participants (eg demographic, clinical, social) and information on exposures and potential confounders	5
		(b) Indicate number of participants with missing data for each variable of interest	5,6
		(c) Summarise follow-up time (eg, average and total amount)	5
Outcome data	15*	Report numbers of outcome events or summary measures over time	5.6
Main results	16	(*a*) Give unadjusted estimates and, if applicable, confounder-adjusted estimates and their precision (eg, 95% confidence interval). Make clear which confounders were adjusted for and why they were included	5,6
		(*b*) Report category boundaries when continuous variables were categorized	4
		(*c*) If relevant, consider translating estimates of relative risk into absolute risk for a meaningful time period	
Other analyses	17	Report other analyses done—eg analyses of subgroups and interactions, and sensitivity analyses	
**Discussion**			
Key results	18	Summarise key results with reference to study objectives	7
Limitations	19	Discuss limitations of the study, taking into account sources of potential bias or imprecision. Discuss both direction and magnitude of any potential bias	8
Interpretation	20	Give a cautious overall interpretation of results considering objectives, limitations, multiplicity of analyses, results from similar studies, and other relevant evidence	7,8
Generalisability	21	Discuss the generalisability (external validity) of the study results	8
**Other information**			
Funding	22	Give the source of funding and the role of the funders for the present study and, if applicable, for the original study on which the present article is based	8

*Give information separately for exposed and unexposed groups.

Note: An Explanation and Elaboration article discusses each checklist item and gives methodological background and published examples of transparent reporting. The STROBE checklist is best used in conjunction with this article (freely available on the Web sites of PLoS Medicine at http://www.plosmedicine.org/, Annals of Internal Medicine at http://www.annals.org/, and Epidemiology at http://www.epidem.com/). Information on the STROBE Initiative is available at http://www.strobe-statement.org.

For peer review only - http://bmjopen.bmj.com/site/about/guidelines.xhtml
